# Evaluation of Triage Tests When Existing Test Capacity Is Constrained: Application to Rapid Diagnostic Testing in COVID-19

**DOI:** 10.1177/0272989X211014114

**Published:** 2021-05-19

**Authors:** Janet Bouttell, Neil Hawkins

**Affiliations:** Health Economics and Health Technology Assessment, University of Glasgow, Glasgow, UK; Health Economics and Health Technology Assessment, University of Glasgow, Glasgow, UK

**Keywords:** diagnostic test, evaluation, triage test, sensitivity, specificity

## Abstract

**Objectives:**

A triage test is used to determine which patients will undergo an existing or “reference” test. This article explores the potential value of using triage tests before reference tests when the capacity of the reference test is constrained.

**Methods:**

We developed a simple model with inputs: prevalence, sensitivity, specificity, and reference test capacity. We included a case study of rapid diagnostic tests for SARS-CoV-2 antigens used as triage tests before a reference polymerase chain reaction test. Performance data were obtained from an evaluation performed by an academic center on 425 samples from testing centers in the United Kingdom and Germany.

**Results:**

When reference test capacity is constrained, the use of a triage test leads to a relative expansion of the population tested and cases identified; both are higher with a high-specificity triage test. When reference test capacity is not constrained, the potential advantages of introducing a triage test can be assessed using a standard cost-utility framework, balancing the utility of the reduction in the number of reference tests required against the disutility of missed cases associated with the use of a lower-sensitivity triage test. In the constrained case, the advantage of a triage testing strategy in terms of population covered and cases identified is reduced as the prevalence increases. In the unconstrained case, the reduction in reference tests required is reduced and the number of cases missed increase as the prevalence rises.

**Conclusion:**

When the availability of the reference test is constrained, tests added in a triage position do not need high levels of accuracy to increase the number of cases diagnosed. This has implications in many disease areas, including COVID-19.

**Highlights**

- Where tests are used in a triage position (to determine who undergoes a high-accuracy diagnostic test), lower-accuracy tests can still be useful.
- When there is limited capacity of the high-accuracy diagnostic test, triage testing strategies can increase the population covered and the number of cases identified.
- When the capacity of the high-accuracy diagnostic test is not limited, triage tests can reduce the number of high-accuracy tests required, although this will be at the cost of missed cases.

The potential value of a test with given performance, in terms of specificity and sensitivity, depends on how the test will be used in clinical practice and the disease prevalence in the population of interest. A new test may be used in a variety of ways. It may be used as a replacement for an existing test, as an add-on to an existing test, or as a triage test. When used as a triage test, the result of the new test determines which patients will then undergo an existing test.^1^ These alternative uses are presented in Figure 1.

**Figure 1:**
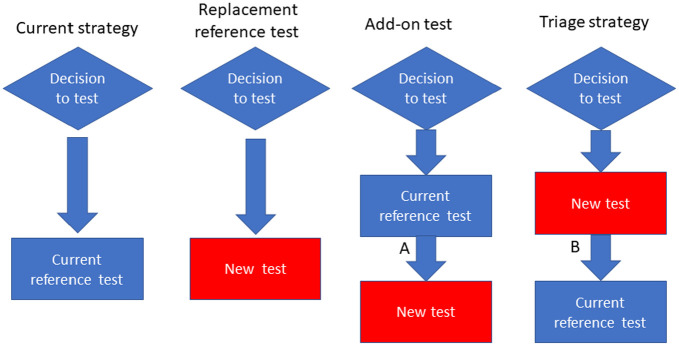
Roles of tests and positions in existing diagnostic pathways.

The introduction of a triage test does not aim to improve the diagnostic accuracy of the current pathway. Rather, it reduces the use of existing tests that may be invasive, cumbersome, or expensive or for which the patient may need to wait for the test, result, or both.^2^ A simple form of triage testing is pooling, in which multiple patient samples are included in 1 sample for testing and individual tests are carried out only if the combined sample tests are positive.^3^ A practical example of a triage test is a blood test measuring levels of D-dimer in patients with pulmonary embolism.^2,4^ The “reference” diagnostic test is computed tomography (CT), which is highly accurate. However, CT is expensive, requires skilled staff to perform it, and its capacity is constrained, so patients may need to wait before they are tested. Although D-dimer has low specificity (about 50%), it has high sensitivity and negative predictive value (greater than 99% at a prevalence of 22%).^4^ This means that although D-dimer does not pick up all patients who are disease negative, when there is a negative result, it is highly likely that the patient is disease negative. Because the test is cheap and there is no waiting time, D-dimer has potential as a triage test to rule out pulmonary embolism.

A further example of a triage testing strategy is in tuberculosis (TB). The existing reference test is problematic in high-burden countries, as it is based on culturing bacteria from sputum samples. This is time-consuming and may miss cases in important patient groups, such as in children and individuals with human immunodeficiency virus as they struggle to produce sputum. The World Health Organization (WHO) set out minimum and optimum test performance targets in a consensus document produced in 2014.^5^ As with D-dimer, the target product profile (TPP) prioritized sensitivity (minimum 90%, optimum 95%) over specificity (minimum 70%, optimum 80%) to minimize false-negatives. A recent article described a blood-based triage test in development that is close to meeting the TPP (sensitivity 86% and specificity 69%) in adult patients with persistent cough.^6^ By way of example, these test performance statistics would translate to a negative predictive value of 97% at 17% prevalence among patients with cough in Southeast Asia.^7^

We can classify triage tests as either “rule-in” tests, which are intended to confirm that a patient has a specific disease, or as “rule-out” tests, which are intended to confirm that a patient does not have a disease. Rule-in tests require high specificity and rule-out tests require high sensitivity. This relationship is known as the SPIN and SNOUT rule (SPecific test when positive rules IN the disease [SPIN] and Sensitive test when Negative rules OUT the disease [SNOUT]).^8^ The extent to which a rule-in or rule-out test is preferred will depend on the prior probability of disease and the consequences, both to the patient and in terms of health care resource use of false-positives and false-negatives. Both the D-dimer test and the TB test described above are rule-out tests with high sensitivity so that negative results are highly predictive of no disease. The rule-out test is useful in this role, as the consequences of a false-negative are that a serious condition may go untreated.

In the context of COVID-19 in the European Union (EU), the WHO currently recommends diagnosis by molecular tests, which detect the ribonucleic acid (RNA) of the SARS-CoV-2 virus.^9^ These tests require well-equipped laboratories, highly skilled technologists, and multiple reagents. Testing capacity has been constrained by infrastructure limitations and supply shortages in the EU^9^ and is highly likely to be constrained in less developed countries around the world.^10^ WHO has called for the development of rapid diagnostic tests (RDTs), which are based on the detection of antigens related to SARS-CoV-2.^11^ Antigen tests aim to detect the presence of viral proteins expressed by the virus in a specimen taken from a person’s respiratory tract. How well these tests work depends on several factors, including the time from onset of symptoms, the quality of the specimen collected from the patient, how the sample is stored, transported, and processed, as well as the design of the test.^11^ Based on experience with other respiratory diseases, WHO estimates that the sensitivity of these tests might be expected to vary from 34% to 80%.^11^ WHO does not currently recommend the use of antigen RDTs for clinical decision making but does recommend further research into antigen-based tests as they have potential diagnostic utility with improved test performance.^11^ When testing resources are constrained, WHO recommends that testing is prioritized for vulnerable patients at risk of serious disease, health care workers, and the first symptomatic individuals in a closed setting such as a prison.^12^

In this article, we set out a method of applying quantitative measures such as sensitivity and specificity, as stated in documents such as WHO TPPs, to particular diseases and local contexts to provide more meaningful metrics to decision makers. We use antigen tests for SARS-CoV-2 as examples to demonstrate that, if facilities for reference testing are restricted, there may be a role for RDTs to be used as triage tests to enrich the prevalence of disease in the population tested using the reference test and improve the efficiency of the laboratory-based RNA testing process. This allows a greater number of cases to be detected than using reference tests (the RNA testing process) alone. We present a simple modeling approach that can be used to explore the use of a triage test prior to a reference test. We contrast 2 situations in which the availability of the reference test is capacity constrained or unconstrained. We show that the relevant metrics of test value, and the value of a test with given characteristics, vary depending on whether the existing reference test capacity is constrained or not. We develop generic models and then apply them in a case study using test data for an RDT for SARS-CoV-2. We describe the limitations of these models and the assumptions that would be required when using their outcomes for decision making. We also discuss their evidential requirements and contrast these with the requirement for full-blown cost-effectiveness models. The models presented in this article are not intended to provide definitive estimates of the clinical value and cost-effectiveness of a test; this typically require the careful identification and synthesis of evidence and the development of detailed decision-analytic or cost-effectiveness models, which requires both time and significant resource. Such models are often highly context specific. Rather, the models presented here are simple models that can be readily used to provide an indication of the potential value of a test while it is under development or during some form of expedited review. Such models are likely to be useful during the development of a test and provide a guide as to whether further investment in the development of a test is warranted and what studies are required to provide sufficient evidence to support the uptake and commercialization of a test. We are not seeking to evaluate a particular test or tests for use in a given scenario; rather, we are presenting a methodological illustration of the different metrics that are relevant in evaluating triage tests dependent on whether access to the reference test is or is not constrained. The point applies across disease areas.

## Methods

A decision-analytic model was developed that predicted true- and false-positive and -negative rates for a triage test (T) with given sensitivity (SENS), specificity (SPEC), and prevalence. In this case, we have defined sensitivity and specificity strictly in terms of the probability that the triage test predicts positive and negative test results for a patient receiving the existing or “reference” test that is used to determine the future treatment of a patient. In this notation, P[R^+^] (probability of testing positive in the reference test) is effectively the prevalence, as we are concerned only with positives and negatives as defined by the reference test. The reference test may not be 100% accurate, but this is a simplifying assumption made for the purposes of this illustration.

$$\mathrm{SENS}=P[T^{+}|R^{+}]$$

$$\mathrm{SPEC}=P[T^{-}|R^{-}]$$

**Figure 2:**
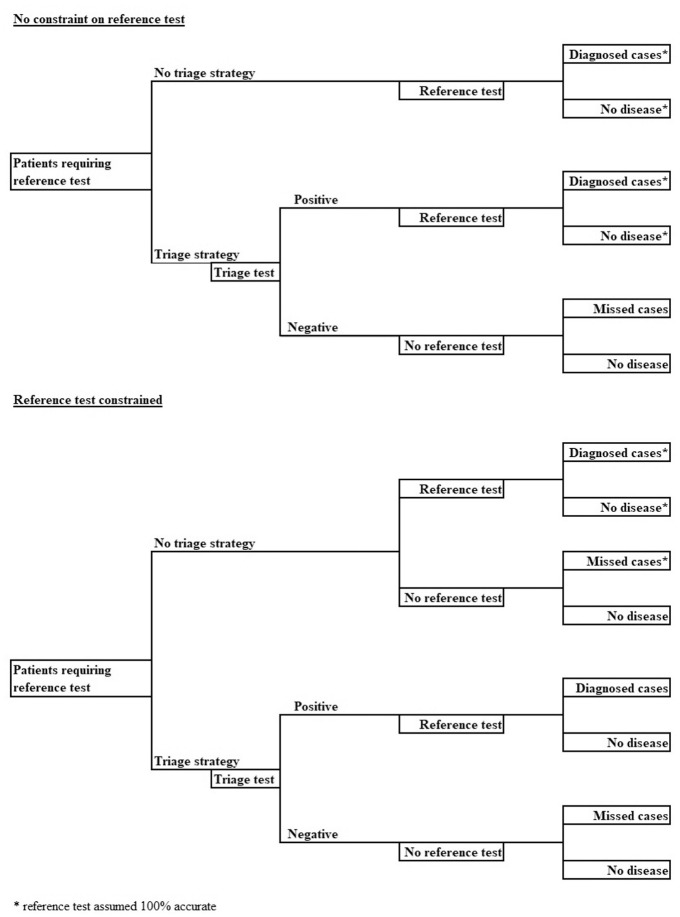
Model predicting true- and false-positive and -negative rates for a triage test.

The model is shown in Figure 2.

We have considered 2 use cases. One in which the reference test capacity is constrained and currently fully used and one in which the reference test capacity can be varied in the short term according to demand. In practice, there may exist a third use case in which capacity can be varied only in the mid to long term.

### Capacity-Constrained Reference Test Use Case

Where the reference capacity is constrained, we can estimate the probability of testing positive at triage testing ($P\left[T^{+}\right]$) as:

$$P\left[T^{+}\right]=P\left[R^{+}\right]\times \mathrm{SENS}+\left(1-P\left[R^{+}\right]\right)\times \left(1-\mathrm{SPEC}\right)$$

This provides an estimate of the proportion of patients for whom reference testing is indicated following the triage test. The number of patients who can actually be tested will depend on the available capacity.

We can calculate the probability of testing positive at the reference test following a positive result in triage testing as

$$P\left[R^{+}|T^{+}\right]=\frac{P\left[R^{+}\right]\times \mathrm{SENS}}{P\left[R^{+}\right]\times \mathrm{SENS}+\left(1-P\left[R^{+}\right]\right)\times \left(1-\mathrm{SPEC}\right)}$$

The difference between these proportions represents patients who would have tested positive under the reference test but were unable to receive the test because of the capacity constraint. It is assumed that $P\left[R^{+}\right]$ is the same for patients who were able to, and not able to, access the reference test.

We can estimate the maximum relative population size ($\delta_{\mathrm{triage}}$) that can be tested using the triage testing before the reference test capacity is exhausted:

$$\delta_{\mathrm{triage}}=\frac{1}{P\left[T^{+}\right]}$$

We can also estimate the maximum effective relative increase in the population ($\delta_{\mathrm{effective}}$) that can be screened using the triage strategy. This is the factor by which the reference test capacity would need to be expanded by for a reference test–only strategy to identify as many cases as the triage strategy.

$$\delta_{\mathrm{effective}}=\frac{P\left[R^{+}|T^{+}\right]}{P\left[R^{+}\right]}$$

It will be smaller than $\delta_{\mathrm{triage}}$, as the triage test will identify some false-positives.

This is the maximum effective expansion that could be achieved if 1) there are no constraints on the availability of the triage tests and 2) there are sufficient potential cases to be tested to exhaust the reference test capacity (i.e., $\delta_{\mathrm{triage}}\;\mathrm{times}\;\mathrm{the}$ reference test capacity).

We can then finally estimate the increase in the probability of identifying a case from the expanded population that arises from the use of the triage test.

$$P\left[R^{+}|T^{+}\right]-P\left[R^{+}\right]$$

This increased number of cases detected can be traded off against the cost of the triage testing.

### Reference Test Capacity Unconstrained Use Case

Where the reference capacity is unconstrained, we can estimate the reduction in the number of reference tests undertaken and the number of false-negative results arising from the use of the triage tests.

The reduction in the proportion of patients requiring the reference test following the triage test (the probability that a patient tests negative on the triage test) is given by

$$1-\left(P\left[R^{+}\right]\times \mathrm{SENS}+\left(1-P\left[R^{+}\right]\right)\times \left(1-\mathrm{SPEC}\right)\right)$$

The proportion of missed cases, patients who test negative on the triage test but would have tested positive on the reference test, is given by

$$P\left[R^{+}\right]\times \left(1-\mathrm{SENS}\right)$$

The reduction in the proportion of patients requiring the reference test and cost savings from that reduction will need to be traded off against the missed cases.

## Case Study

We applied this model to an exemplar antigen test from the 137 listed on February 5, 2021, by the nongovernmental organization, the Foundation for Innovative New Diagnostics (FIND)^13^ (see Table 1). FIND is a global, nonprofit organization funded primarily by donations from international governments and aid foundations. Its aim is to accelerate the development of diagnostics in diseases of poverty.^13^ During the COVID-19 pandemic, FIND acted as a register of tests in development and has commissioned independent evaluations of some tests.^13^ All tests evaluated by FIND to date have relatively high specificity. For illustrative purposes, we chose an evaluated test with relatively low sensitivity (Coris BioConcept COVID-19 Ag Respi-Strip^14^) and then contrasted its performance with a hypothetical test with the sensitivity and specificity of the test reversed.

**Table 1:** Rapid Diagnostic Tests for SARS-CoV-2: Case Study

Test Type	Characteristic	Exemplar Test	Sensitivity	Specificity
Rule-in	High specificity	Coris BioConcept test^14^	50%	95.9%
Rule-out	High sensitivity	Inverse of above	95.9%	50%

The Coris BioConcept test^14^ is a CE-marked antigen test, meaning it conforms with the relevant EU legislation, directive 98/79/EC, on in vitro diagnostic tests. Test performance was taken from the external evaluation report for the test published by FIND on December 10, 2020.^14^ Data for the evaluation were collected from 3 sites in the United Kingdom and Germany from May to August 2020.^14^ The samples were taken from hospitalized patients in the United Kingdom and from patients suspected as having SARS-CoV-2 infection at a walk-in center in Germany.^14^ The prevalence of SARS-CoV-2 infection among the study population was just less than 2%.^14^ For the purposes of the case study, we have selected a base-case prevalence of 5% based on the WHO estimate of prevalence in a symptomatic general population and contacts of an index case.^15^ Sensitivity analysis was conducted, showing the incremental probability of detection with a triage strategy for the constrained-use case, proportionate reduction in reference case use in the unconstrained-use case, and the false-positive rate for a full range of prevalence (reference test positive) rates. Contour plots are presented showing the maximum relative expansion of reference test coverage in the constrained-use case and the proportionate reduction in reference testing at different levels of sensitivity and specificity (holding prevalence constant at 5%).

There was no specific funding for this study.

## Results

Table 2 provides the probability of a positive test at triage for rule-in and rule-out triage strategies, illustrating that the probability of a positive reference test after triage is greatest with a rule-in test (high specificity) at 39.1% as compared with the probability of a positive reference test after triage with a rule-out test at 9.2%. Both triage strategies increase the probability of a positive reference test by enriching the prevalence in the population taking the reference test. Table 3 illustrates the clinical impact of the triage strategies for the constrained-use case and Table 4 for the unconstrained use case. Different metrics are relevant in each use case. Table 3 shows that where reference test capacity is constrained, the relative expansion of the population and the number of cases identified is greater with the higher-specificity, rule-in test. Although the number of false-negatives at triage is also greater with the rule-in test because of the lower sensitivity, this does not equate to missed cases, because the total number of cases identified is still higher with the rule-in test because of the expansion of the tested population. For the unconstrained reference capacity case (see Table 4), the higher-specificity rule-in test leads to the greatest reduction in the number of reference tests required; however, the rule-in test also leads to the greatest number of missed cases. The higher sensitivity rule-out test minimizes the number of missed cases but is less efficient in reducing the number of reference tests.

**Table 2:** Results of Triage Testing^a^

Strategy Parameter	Triage Test + Reference Test	
	Rule-in Test (Sensitivity 50.0%, Specificity 95.9%)	Rule-out Test (Sensitivity 95.9%, Specificity 50.0%)
Probability of positive test at triage	6.4%	52.2%
Probability of false-negatives at triage	2.5%	0.2%
Probability of positive reference test	39.1%	9.2%

a The probability of a reference test being positive under a reference test–only strategy is equivalent to prevalence (5% in the base case).

**Table 3:** Clinical Impact Where Reference Test Capacity Is Constrained (Prevalence 5%)

Strategy Parameter	Triage Test + Reference Test	
	Rule-in Test (Sensitivity 50.0%, Specificity 95.9%)	Rule-out Test (Sensitivity 95.9%, Specificity 50.0%)
Relative size of triage population needed to exhaust reference test capacity	15.6	1.9
Maximum effective relative expansion of reference test coverage (the expansion of the population needed to exhaust reference test capacity)	7.8	1.8
Incremental probability of a positive reference test	34.1%	4.2%

**Table 4:** Clinical Impact Where Reference Test Capacity Is Unconstrained (Prevalence 5%)

Strategy Parameter	Reference Test Only	Triage + Reference Test	
		Rule-in Test (Sensitivity 50.0%, Specificity 95.9%)	Rule-out Test (Sensitivity 95.9%, Specificity 50.0%)
Reduction in reference testing	—	93.6%	47.7%
Probability of a missed case	0	2.5%	0.2%

**Figure 3:**
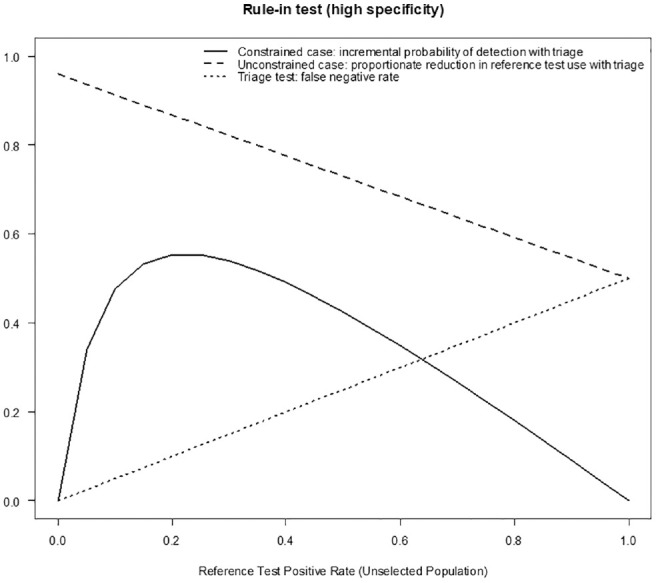
Incremental probability of detection with a triage strategy when reference tests are constrained, proportionate reduction in reference test use when reference tests are unconstrained and triage test false-negative rate for rule-in triage test (high specificity of 95.9% and sensitivity of 50%) at different levels of prevalence (reference test positive).

**Figure 4:**
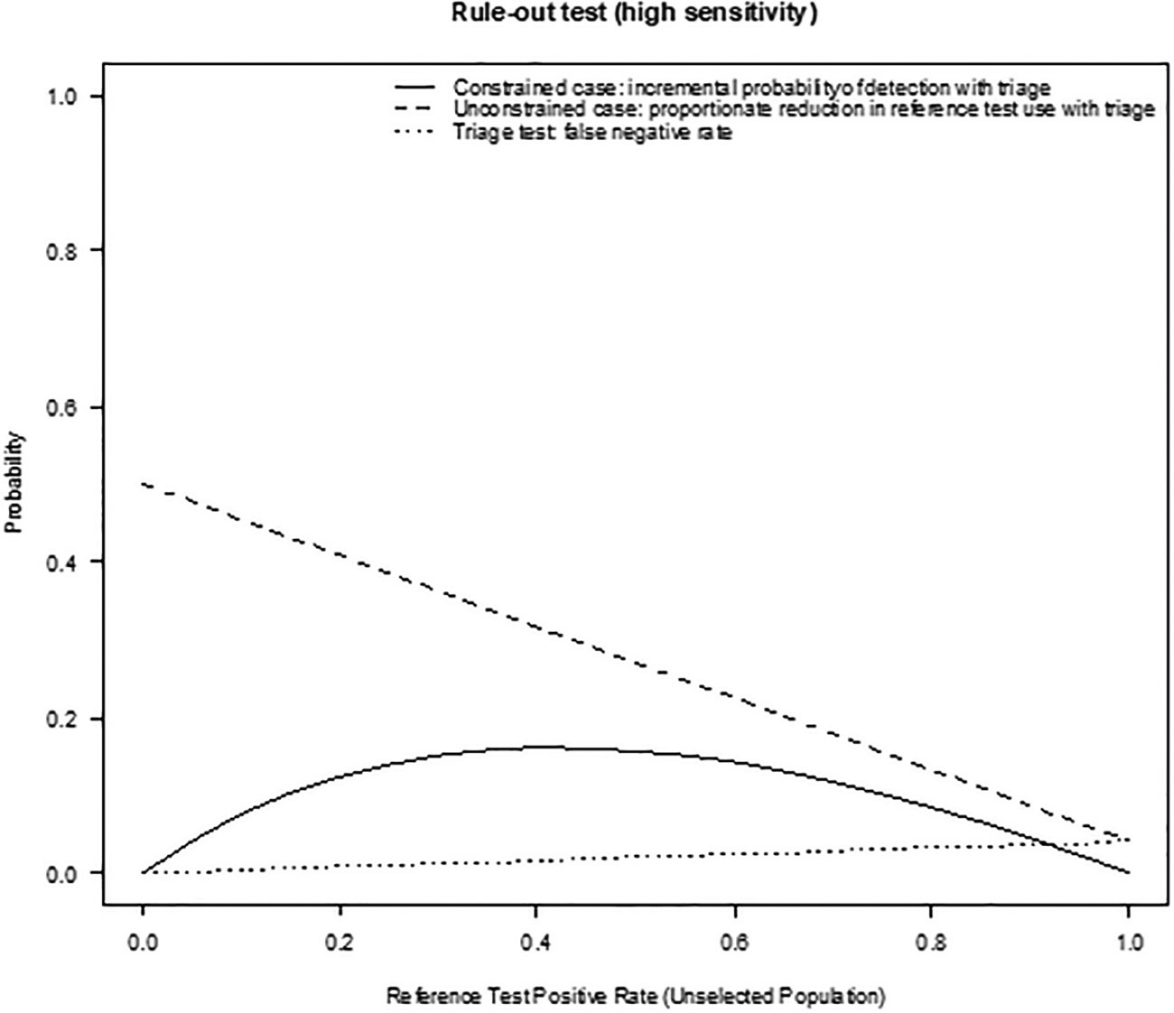
Incremental probability of detection with a triage strategy when reference tests are constrained, proportionate reduction in reference test use when reference tests are unconstrained and the triage test false negative rate for rule-out triage test (high sensitivity 95.9% and specificity of 50%) at different levels of prevalence (reference test positive).

**Figure 5:**
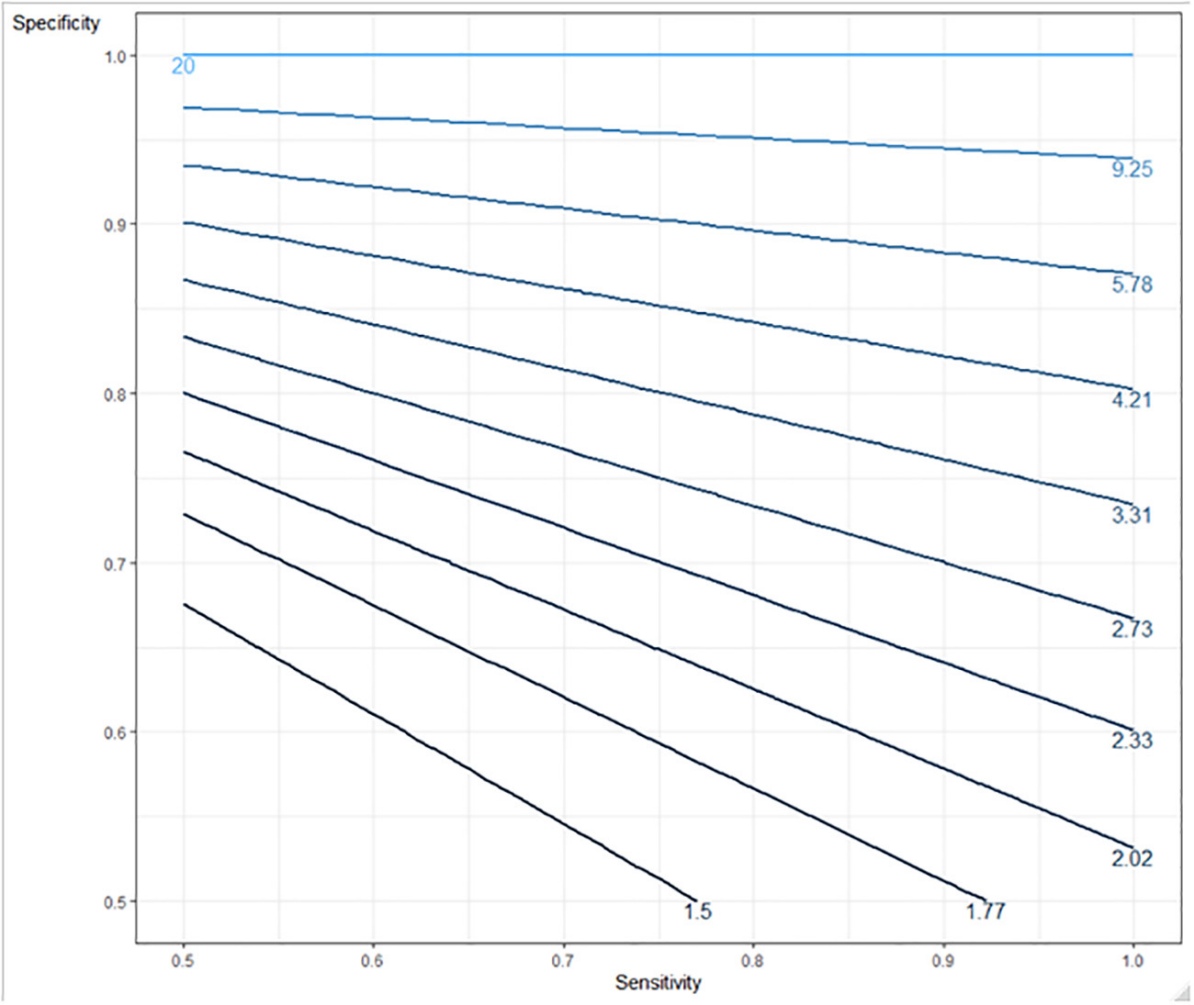
Contour plot showing the maximum effective relative expansion of reference test coverage in the constrained case as a function of sensitivity and specificity (5% reference test–positive rate in an unselected population).

**Figure 6:**
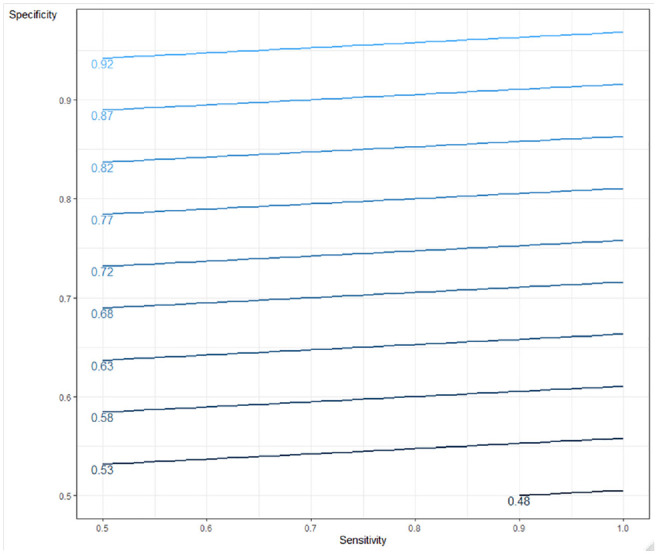
Contour plot showing the proportionate reduction in reference testing in the unconstrained case as a function of sensitivity and specificity (5% reference test–positive rate in an unselected population).

Sensitivity analysis compared the results shown in Tables 2 and 3 (using a base-case prevalence of 5%) for the full range of prevalence. Figures 3 and 4 show the incremental probability of detection with a triage strategy when reference tests are constrained, the proportionate reduction in reference test use when reference tests are unconstrained, and the triage test false-positive rates for rule-in and rule-out tests, respectively, at different levels of prevalence (reference test positive). In the constrained case, there is a greater probability of cases being identified using a triage strategy regardless of the increasing prevalence with our example of a rule-in triage test outperforming the rule-out test because of higher specificity. In the unconstrained case, a triage testing strategy offers a reduction in the reference tests required (and corresponding cost), although this benefit is reduced as the prevalence increases and more confirmatory reference tests are required. Again, the rule-in test outperforms the rule-out test. The benefit from the reduction in reference tests required must be offset against missed cases, which increase as the prevalence rates rise. The rule-out test example demonstrates a smaller increase, as it is more sensitive. Figure 5 provides a contour plot showing the maximum relative expansion of the reference test population in the constrained-use case for different levels of sensitivity and specificity. The plot demonstrates that the expansion of the population needed to exhaust the reference test capacity in the constrained case varies from 1.5 to 20, depending on the specificity and sensitivity of the test. By way of example, with specificity at 80% and sensitivity at 50%, 2.33 times the number of people could be tested using a triage strategy rather than a reference test–only strategy. With a reference test capacity of 1000, 2330 people could be tested under the triage strategy. Figure 6 is a further contour plot showing the proportionate reduction in reference tests required in the unconstrained-use case for different levels of sensitivity and specificity. As a result of introducing triage tests in an unconstrained-use case, the number of reference tests required can be reduced. The proportionate reduction depends on the sensitivity and specificity of the test, as illustrated by Figure 6. By way of example, at 80% sensitivity and 80% specificity, the reduction in reference tests required is 0.77. If 1000 reference tests are required under the reference test–only strategy, this will be reduced to 230 under a triage strategy, a reduction of 770 reference tests. Both plots assume a prevalence level (reference test positive) of 5%. The contour plots demonstrate the relative importance of specificity over sensitivity in delivering higher population coverage when reference tests are constrained and a higher reduction in reference tests required in the unconstrained-use case. In the unconstrained-use case, lower sensitivity will result in a higher level of missed cases.

## Discussion

Where the reference test capacity is constrained and triage tests are unconstrained, the assessment of the opportunity cost of a triage strategy depends on the cost of the triage testing and an assessment of the value of the additional cases identified. The number of additional cases identified is maximized by maximizing specificity. Arguably, sensitivity is less important, as false-negatives at triage testing do not directly represent missed cases. As long as there is a sufficient pool of individuals who would not be tested under the reference test–only regime, the total number of cases identified will be greater under the triage strategy. However, there still may be a cost associated will false-negatives at triage testing if these lead to a change in behavior in those who test negative at triage.

Where the reference test capacity is unconstrained, the assessment of the opportunity cost of a triage strategy depends on the cost of the triage testing, the costs resulting from a reduction in reference testing, and the cost of any false-negatives at triage testing in terms of worse outcomes. The choice of test will depend on the tradeoff between cost savings and missed cases due to false-negatives at triage.

When the availability of the gold standard diagnostic test is constrained, tests added to the diagnostic pathway in a triage position do not necessarily need high levels of accuracy to increase the number of cases diagnosed. This has implications across a wide range of disease areas and is of particular importance during the current COVID-19 pandemic, as decision makers may be able to make use of a wider range of tests, including RDTs, in designing testing strategies where the availability of laboratory-based molecular testing is limited. Our model provides a simple way of assessing whether a particular test may have the potential to increase diagnoses.

Our study has shown that the value of a triage test, in terms of additional cases diagnosed, depends on whether the availability of the gold standard (or reference) test is constrained. Where resource is not constrained, a triage test will result in missed cases and an overall reduction in diagnoses unless its accuracy is high. However, where the availability of the reference test is constrained, there is the potential for tests with relatively low accuracy to improve the levels of diagnosis and reduce missed cases. The levels of test performance required depend on the extent to which the capacity of the reference test is constrained. Levels of test performance can be lower and still deliver benefits as the number of reference tests available is reduced as a proportion of the population to be tested. This finding has relevance across a wide range of diseases and settings. For example, in TB, approximately 3.6 million cases of active disease go undiagnosed each year, partly because of the limited access to confirmatory molecular tests.^16^ A further example is in colorectal cancer. In the United Kingdom, demand for colonoscopy is forecast to increase 10% to 15% per year, resulting in capacity constraints, and fecal immunochemical tests have been suggested as a possible triage test in symptomatic patients.^17^

In relation to the COVID-19 pandemic, testing resources have been constrained in many jurisdictions. Required performance levels for RDTs have been set sufficiently high (for example, the Medicines and Healthcare Products Regulatory Agency [MHRA] in the United Kingdom has set desired performance standards of 97% sensitivity and 99% specificity for tests used to aid in the triage of current SARS-CoV-2 infection by detection of RNA or antigens in samples from people of all ages at any point during active infection^18^) that test manufacturers have failed to meet these levels,^19^ and some tests have been returned to the manufacturers.^20^ It has been suggested that there is a need to be creative in devising a testing strategy.^3,21–23^ Our findings suggest that there may be scope to use tests with lower performance in some testing pathways, although each situation would need to be assessed on its own merits, with the strategy tailored to the current stage of the outbreak and transmission rate in the testing area.

The evaluation of alternative testing strategies using cost-effectiveness analysis typically requires a complex model with parameter estimates for health outcomes and resource use. This resource-intensive process takes time and expertise, and the results may be difficult to generalize, as diagnostic and clinical pathways vary across and within jurisdictions. The model presented is not intended to be an alternative to full-blown cost-effectiveness analysis, as it does not include costs or the health implications of false-positives and -negatives at either the triage or reference test stage and makes the simplifying assumption that the reference test is 100% accurate. Rather, the model is intended to be used during the development of a test to determine whether further investment is appropriate and to guide the design and evidence-generation strategy. However, a potential further use of the simple model may be to inform decision makers responding to infectious disease, such as the current COVID-19 pandemic, who need to evaluate a large number of tests in a wide range of testing scenarios. The model could help to narrow the range of alternatives to be explored in more detailed modeling.

As far as we are aware, this is the first simple model to demonstrate the benefit of triage tests when the availability of reference tests is constrained. The only inputs required for our model are prevalence, sensitivity and specificity, population, and number of reference tests available. Assuming that the incremental net (accounting for resource use) health benefit for treated positive cases compared with untreated positive cases is greater than the incremental net health benefit for treated negative cases compared with untreated negative cases, maximizing the total number of true-positive reference tests will maximize the net health benefit. In general, treated negative cases will be associated with a negative incremental health benefit, as they will be associated with wasted resources and sometimes harm to patients because of unnecessary treatment.
